# Supplementary material for: Evidence for the Desmosomal Cadherin Desmoglein-3 in Regulating YAP and Phospho-YAP in Keratinocyte Responses to Mechanical Forces
Source: Int J Mol Sci. 2019 Dec 10;20(24):6221. doi: 10.3390/ijms20246221 (PMC6940936; doi:10.3390/ijms20246221)
Supplement: Supplementary file 1 [file ijms-20-06221-s001.pdf]

Supplementary materials

Supplementary Table 1. Primer sequences for all genes analyzed in the study

	Gene	Product size (bp)	Forward primer	Reverse primer
Control	POLR2A	73	GCAAATTCACCAAGAGAGACG	CACGTCGACAGGAACATCAG
Target genes	DSG2	211	GAAGCAAGAGATGGCAATGG	GGTTCTGATAATTGGCTGGC
	DSG3	164	CCGAATCTCTGGAGTGGGAA	GCCCAAGGACTAGATGTAGA
	DSC2	207	CCACTTGCCAACATTTACTCG	GCCAAAACCAATGAAGGAGT
	DSC3	207	GTACAAGACATGGATGGCCA	CACTGCCAATTGGAGAGTCA
	DSP	221	GCAGTCTACTGAAGCATACC	CGTAACCCAGACTACAGAAG
	JUP	305	GGCCATTGTGCATCTCATCA	CTGCTCGCCATCTTCAAGTC
	PKP1	249	CAGTTGATTGGGCTGAAGGA	CTACACTGTGAGGAACCTGA
	PKP2	238	GCTACTTTCATACAGCACGAGTG	GAAGCAAACCAGAGACTTGG
	CDH1	96	GCCAGACACATTTATGGAACAG	GTGGAAATGGCACCAGTGT
	CTNNA1	130	GCCTACCTGCAACGCATC	CCTGGATCAGGGACATGG
	CTNNB1	137	TTGGATATCGCCAGGATGAT	CCCATCAACTGGATAGTCAGC
	CCNA2	108	CCATACCTCAAGTATTTGCCATC	TCCAGTCTTTCGTATTAATGATTCAG
	CCNB1	102	CATGGTGCACTTTCCTCCTT	AGGTAATGTTGTAGAGTTGGTGTCC
	CENPA	104	GCACCCAGTGTTTCTGTCAGT	CCAGACAGCATCGCAGAAT
	FOXM1B	279	CCAGGTGTTTAAGCAGCAGA	TCCTCAGCTAGCAGCACCTTG
	MYC	138	CACCAGCAGCGACTCTGA	CTGTGAGGAGGTTTGCTGTG
	NEK2	90	CATTGGCACAGGCTCCTAC	GAGCCATAGTCAAGTTCTTTCCA
	BIRC5	104	AGAACTGGCCCTTCTTGGA	ACACTGGGCCAAGTCTGG
	TOP2A	96	CAGTGAAGAAGACAGCAGCAAA	AAGCTGGATCCCTTTTAGTTCC
	YAP1	83	CCCAGATGAACGTCACAGC	GATTCTCTGGTTCATGGCTGA
	CYR61	190	GAGTGGGTGTGTGACGAGGAT	GGTTGTATAGGATGCGAGGCT
	CTGF	249	CGACTGGAAGACACGTTTGG	AGGCTTGGAGATTTTGGGAG

The primer sequences for *CYR61* and *CTGF* is from [1].

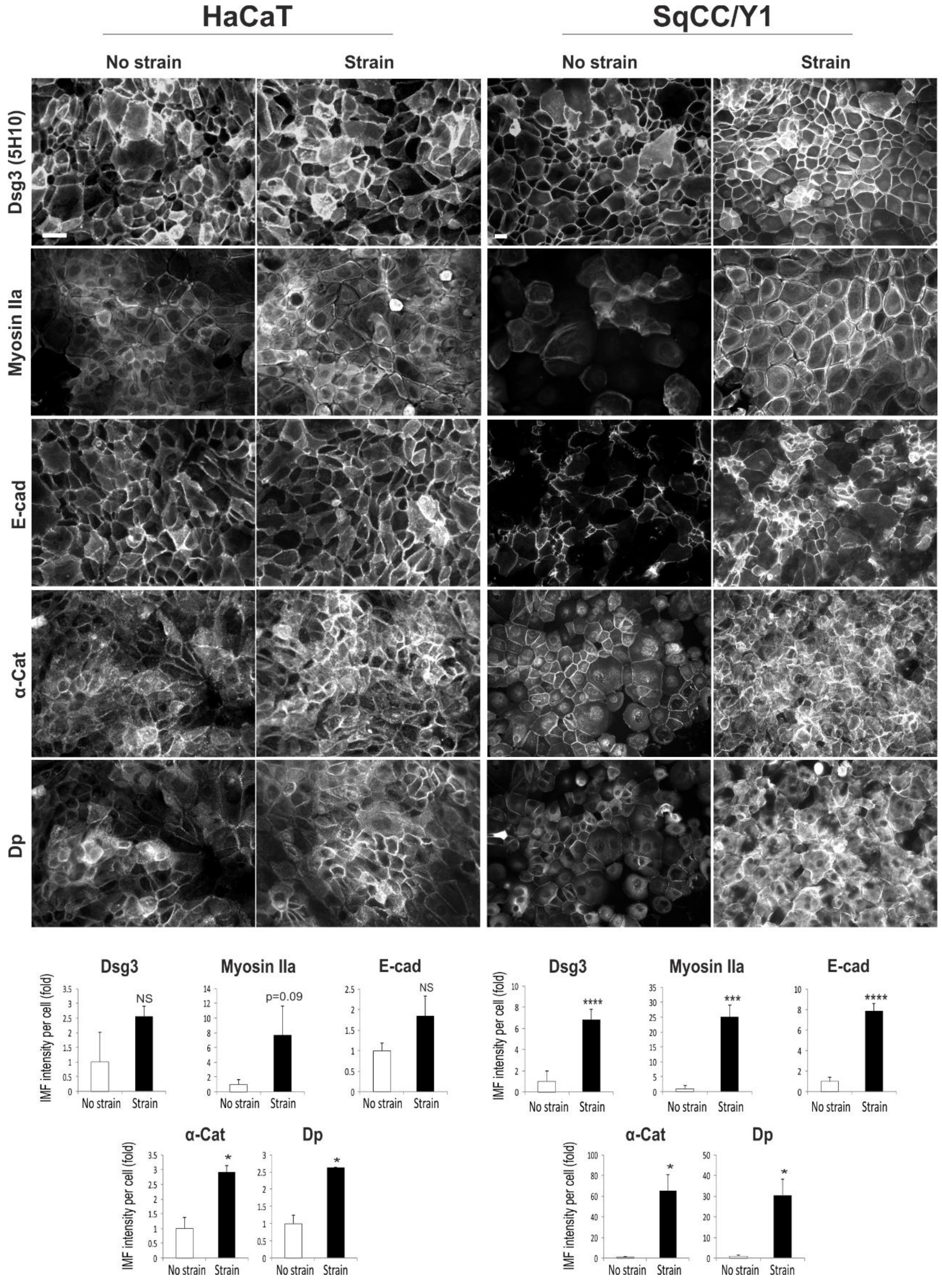

FIGURE S1. Comparison of the peripheral protein expression between skin-derived and oral mucosa-derived keratinocytes in response to cyclic strain. Epi-fluorescent microscopy of HaCaT and SqCC/Y1 cells seeded at confluent densities in Flexcell wells for overnight and then subjected to cyclic strain, or no strain, for 6 hours before fixation with formaldehyde followed by immunostaining for the indicated junctional proteins. Quantitation for each protein in both cell lines are displayed underneath of the images (n=4, mean ± S.D., NS: no significance; *p<0.05, ***p<0.001, ****p<0.0001). Scale bars are 20 μm.

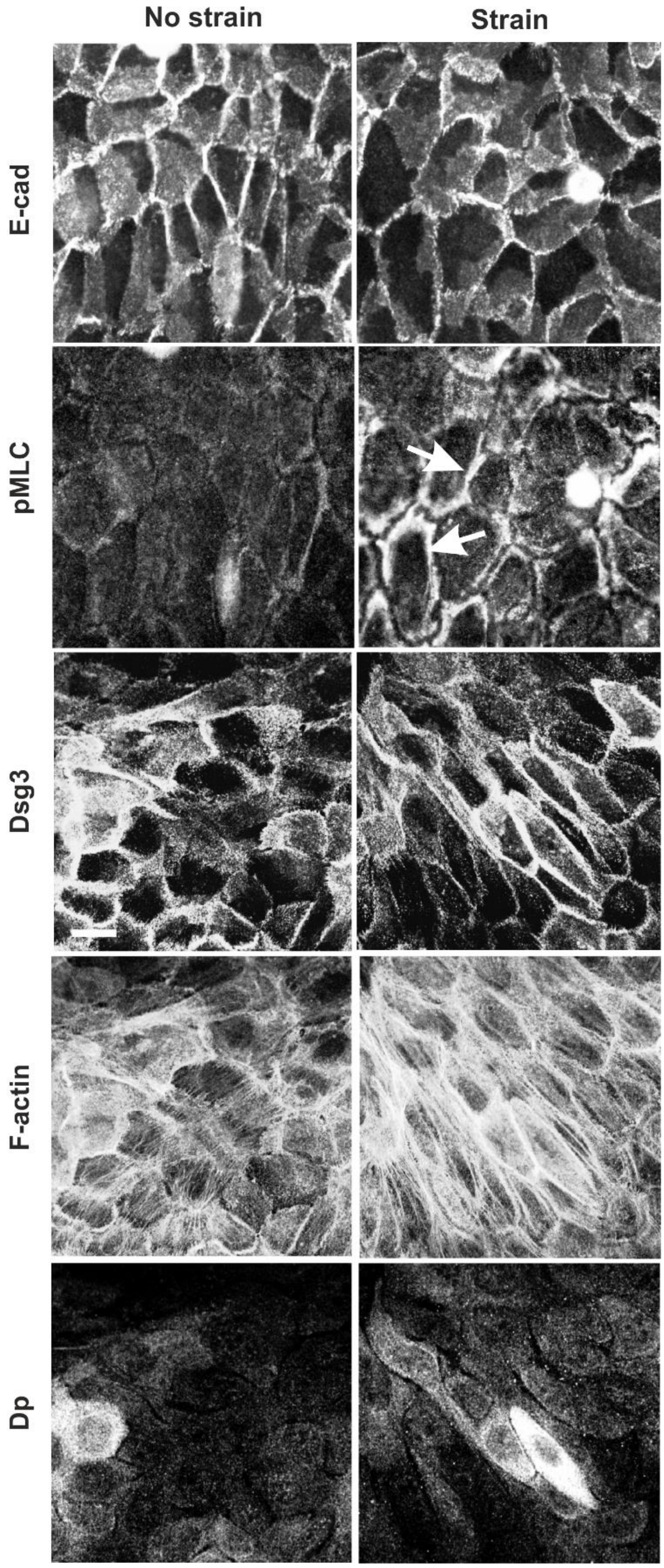

FIGURE S2. Enhanced pMLC expression in HaCaT cells in response to cyclic strain. Confocal microscopy of cells seeded at confluent density in Flexcell wells and subjected to cyclic strain, or no strain, for 6 hours before fixation for total protein analysis (+Triton) of the indicated proteins. Arrows indicate enhanced pMLC at the periphery of strained cells. Evident cortical F-actin bundles were shown in strained cells compared to non-strained cells. Scale bar is 10 μm.

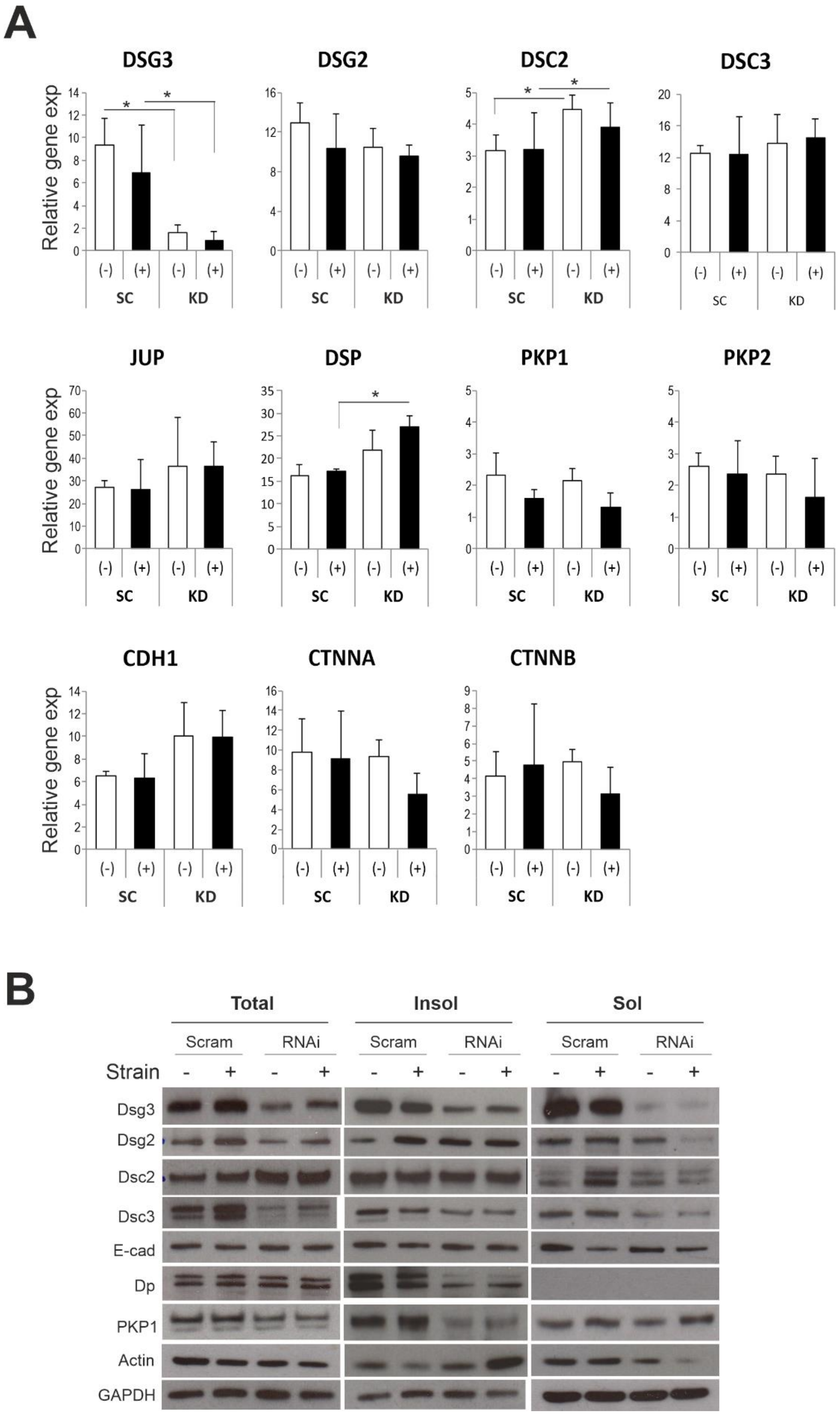

FIGURE S3. RT-qPCR and Western blotting analyses of various junctional components in siRNA pre-treated HaCaT cells subjected to strain, or no strain, for 4 hours. *A*, Pooled data from two independent experiments in HaCaT cells with siRNA transfection (n>5, mean ± S.D., *p<0.05). Overall, only *DSC2* showed a significant increase in the Dsg3 knockdown cells relative to control siRNA treated cells regardless of the mechanical loading. *DSP*, however, showed an increase in response to cyclic strain in

cells with Dsg3 depletion. Similar findings were also observed in samples with cyclic strain for 24 hours (data not shown). *B*, Western blotting analysis of total and Triton soluble/insoluble fractions in siRNA pre-treated HaCaT cells and lysates were extracted immediately after cyclic strain for 6 hours or with no strain. Some variations were observed in different preparations. However, consistently, the residual Dsg3 in Dsg3 depleted cells still showed an increase in response to strain and PKP1 exhibited a reduction in RNA interference (RNAi) treated cells with no response to mechanical loading.

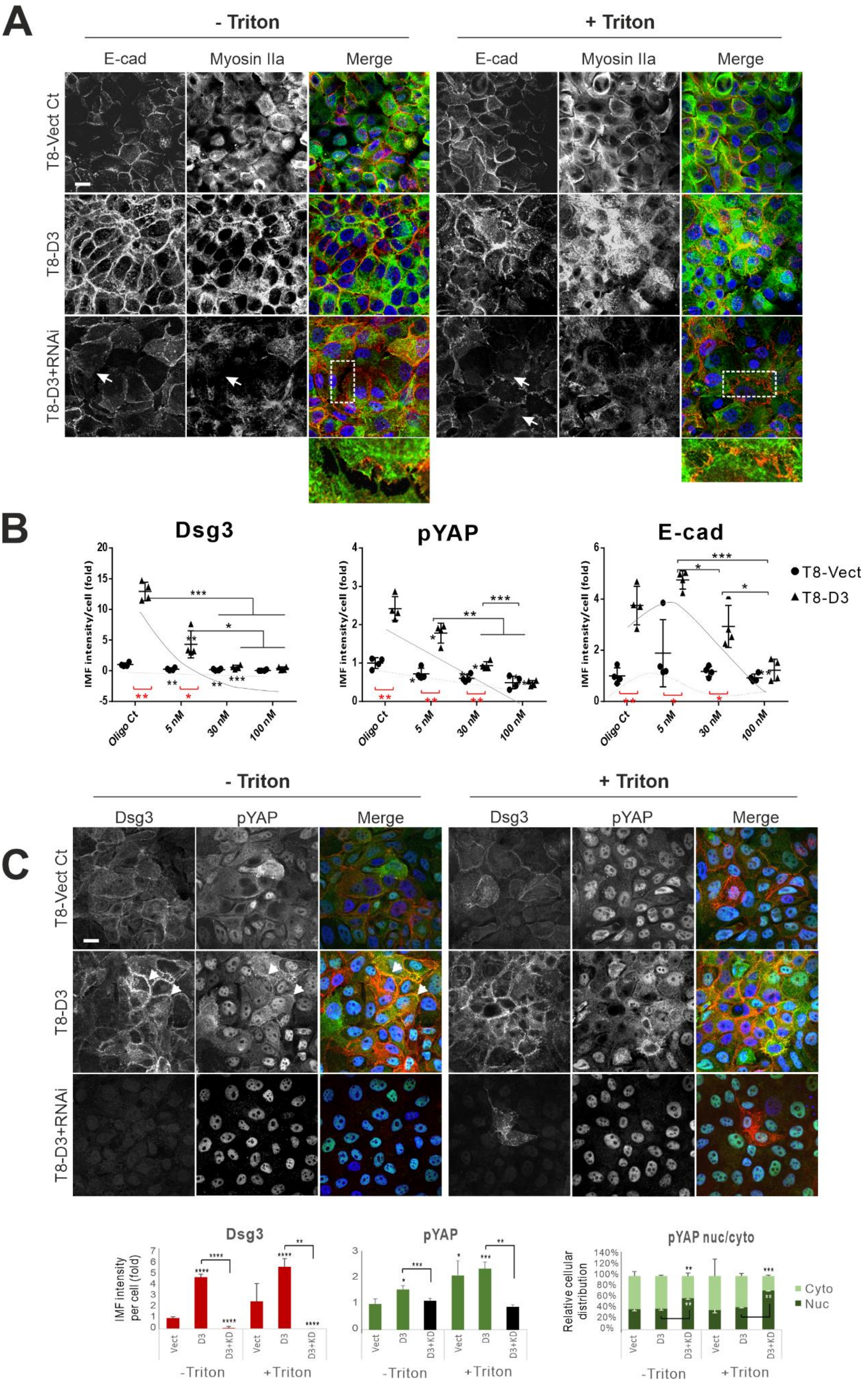
A
- Triton
+ Triton
E-cad
Myosin IIa
Merge
T8-Vect Ct
T8-D3
T8-D3+RNAi
B
Dsg3
pYAP
E-cad
IMF intensity/cell (fold)
Oligo Ct
5 nM
30 nM
100 nM
T8-Vect
T8-D3
C
Dsg3
pYAP
Merge
pYAP nuc/cyto
IMF intensity per cell (fold)
Relative cellular distribution
Vect
D3
D3+KD
-Triton
+Triton
Cyto
Nuc

FIGURE S4. Overexpression of Dsg3 enhances E-cadherin and pYAP expression at the borders with its depletion resulting in marked pYAP nuclear relocation. (**A**) Stable cell lines in cutaneous T8 keratinocytes with transduction of Dsg3 (T8-D3) with or without transient siRNA transfection, alongside vector control (T8-Vect) with relatively low endogenous Dsg3 level. Cells were fixed without (-Triton) or with Triton (+Triton) prior to immunostaining for E-cadherin (red) and Myosin IIa (green) staining. Bottom inserts show the zoomed view of the white marked area highlighting AJ disruption as indicated by arrows in T8-D3+RNAi samples. Note an increase in junctional E-cadherin and Myosin IIa in protein staining of cells/–Triton, but less in sample/+Triton. (**B**) Titration of Dsg3 siRNA in T8-Vect and D3 cell lines by immunofluorescence. The siRNA pretreated cells were seeded on coverslips for 1 day before fixation and immunostaining for the indicated proteins. Only the image quantitation data are shown here (n=4 fields/>350 cells were analyzed per sample). (**C**) Confocal images of cells fixed without (-Triton) and with (+Triton) permeabilization prior to staining for Dsg3 (red) and pYAP (green) in T8 cell lines alongside T8-D3 cells with Dsg3 knockdown (T8-D3+RNAi). Arrows indicate colocalization of Dsg3 and pYAP in T8-D3 cells. Dsg3 knockdown caused pYAP nuclear localization. The image quantitation data were shown below (n=6, mean ± S.D.). (*p<0.05, **p<0.01, ***p<0.001, ****p<0.0001). Scale bar is 10 µm.

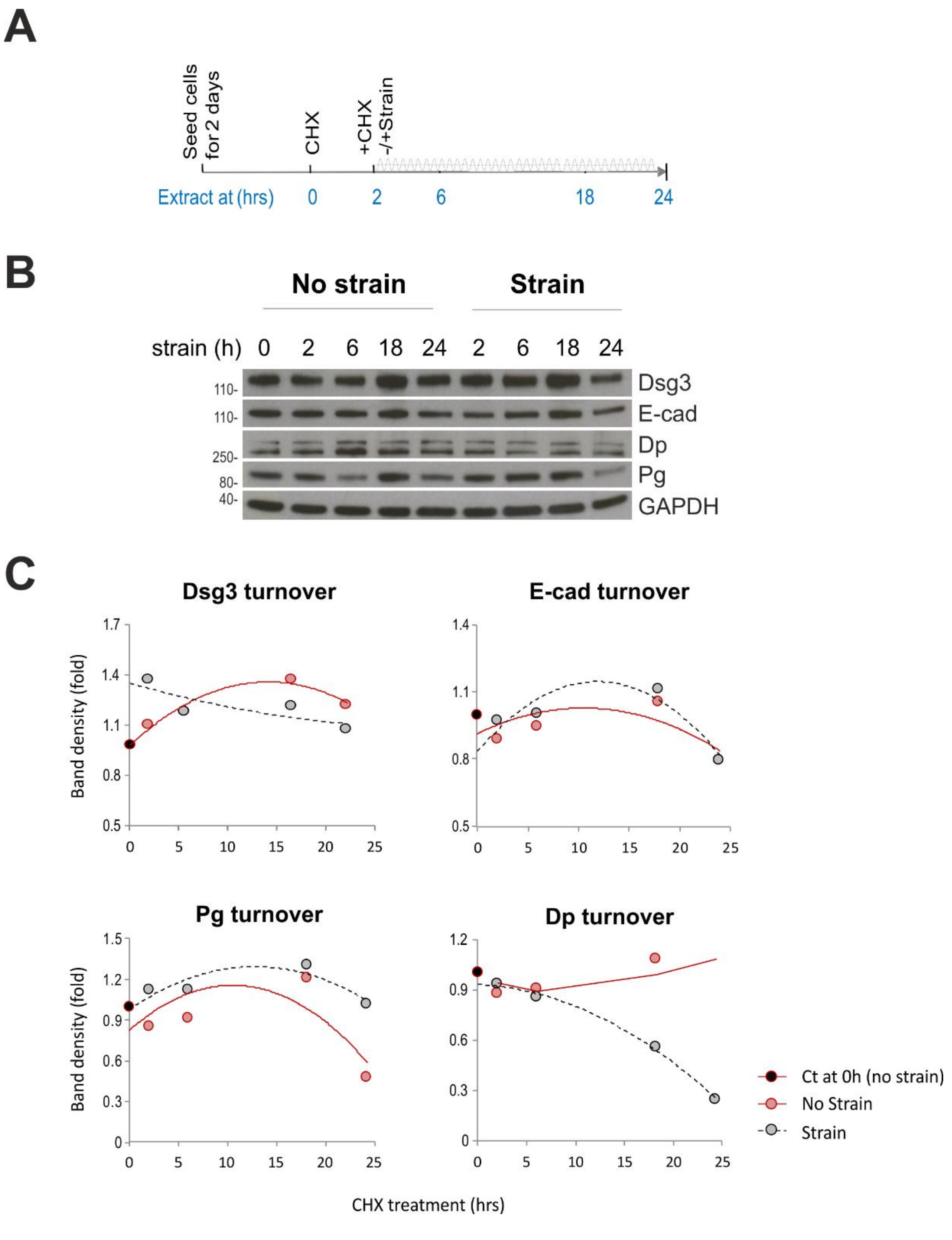

FIGURE S5. Protein turnover analysis in HaCaT cellular response to cyclic strain. *A*, Timeline of the experiment. Two hours before cyclic strain, cycloheximide (CHX, 3 µg/ml) was added into the culture medium and cells with/without strain were treated in the presence of CHX till the end of the experiment (24 hours). *B*, Western blotting for the indicated proteins. GAPDH was used as a loading control. *C*, Quantitation of blots shown in *B*. All bands were normalized against the non-strained sample at the 0 hour time point.

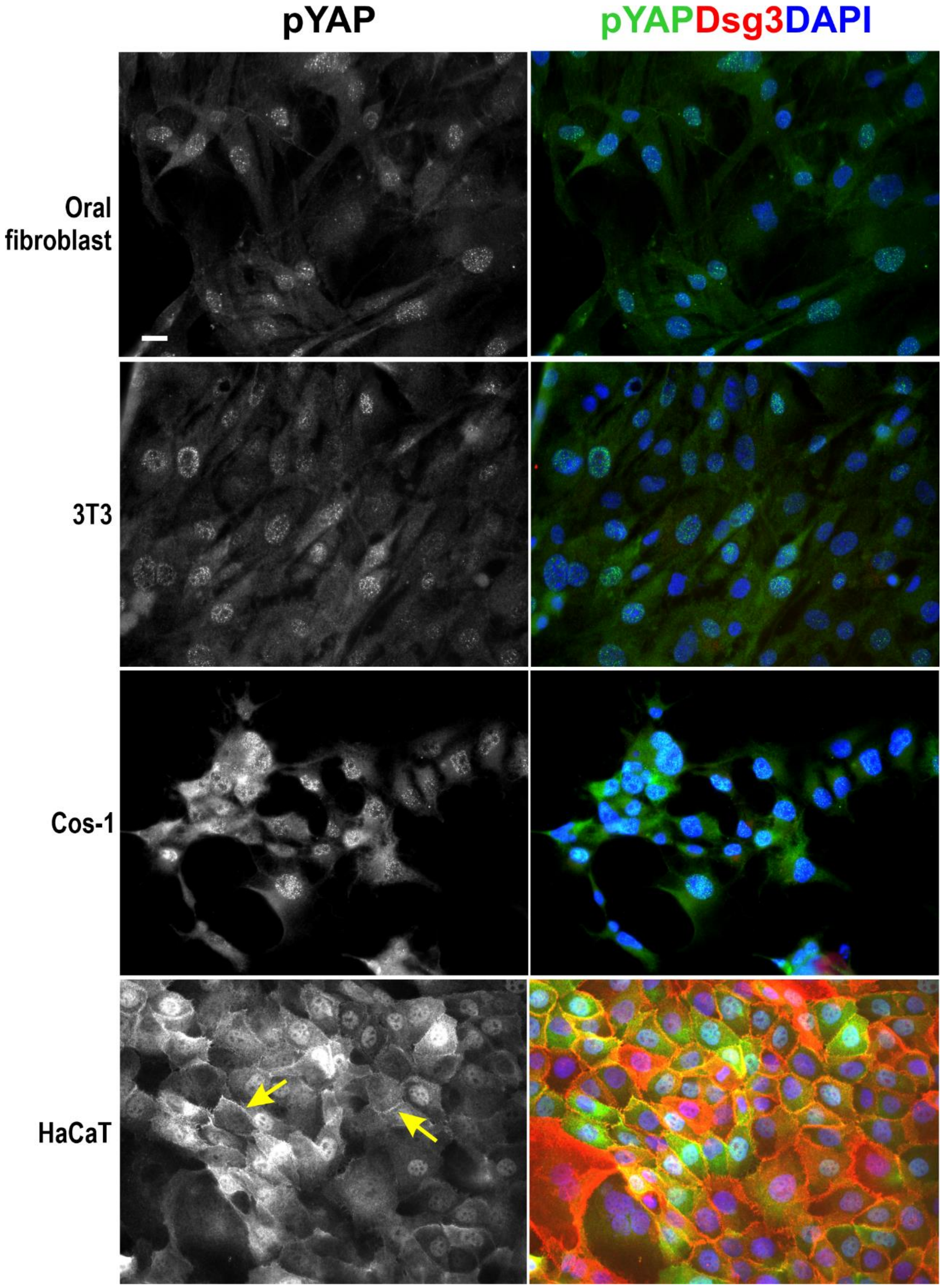

FIGURE S6. Membrane localization of pYAP was found exclusively in NTERT keratinocytes. Fluorescent microscopy of various cell types double-labeled for pYAP (green) and Dsg3 (red) with counter nuclear DAPI staining. Cells were fixed with formaldehyde only prior to immunostaining for the indicated proteins. Note that the membrane pYAP staining (yellow arrows) was detected exclusively in NTERT cells but not in other fibroblast-like cell types. In addition, the level of pYAP expression in NTERT cells appeared greater than other cell types. Scale bar is 10 μm.

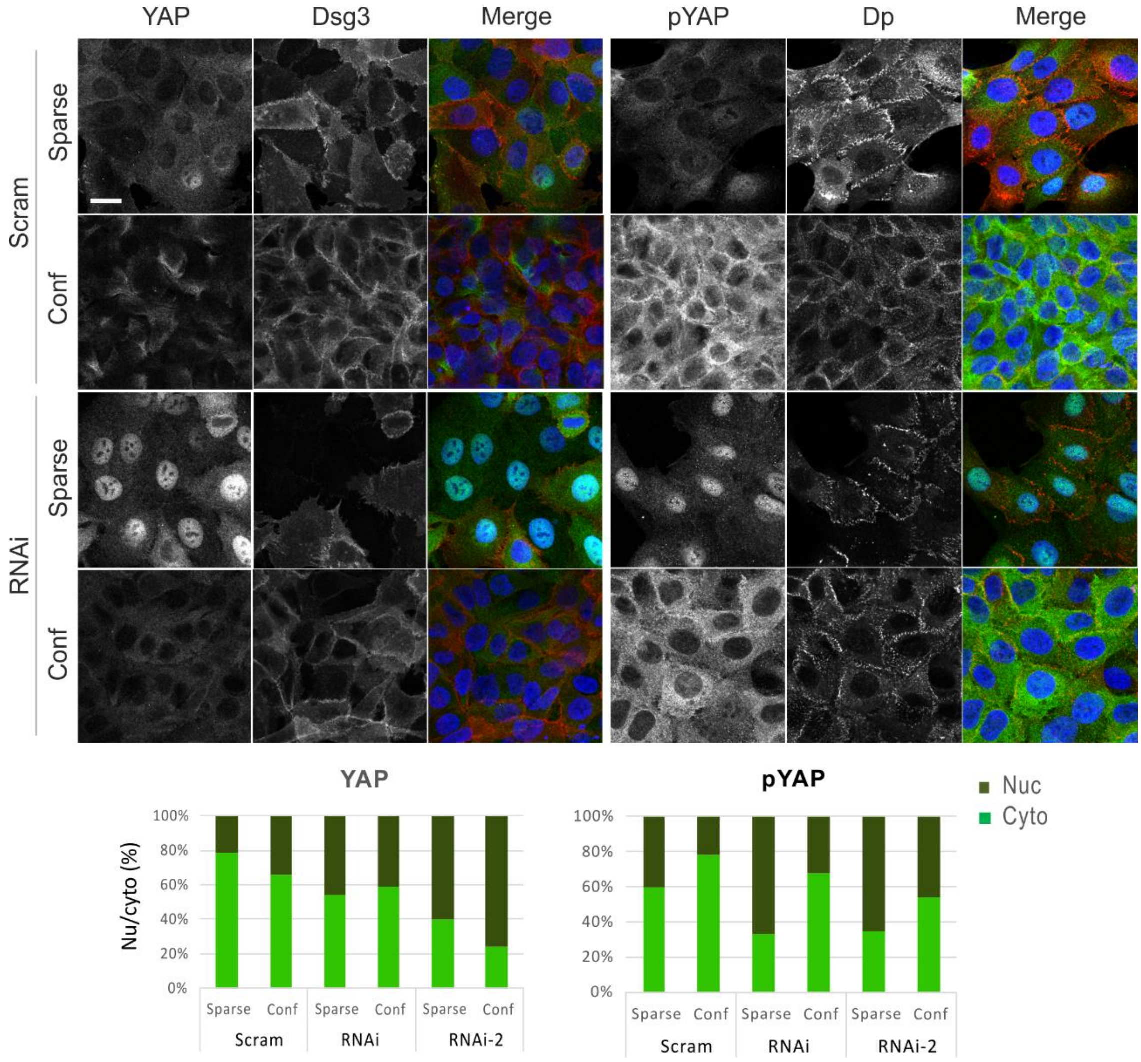

FIGURE S7. Dsg3 knockdown affects YAP/pYAP nuclear exclusion at low cell densities. Confocal images of YAP (green)/Dsg3 (red) (left panels) and pYAP (green)/Dp (red) (right panels) staining in HaCaT cells without (Scram) and with Dsg3 knockdown (RNAi). Cells were seeded at sparse and confluent densities, respectively. Two siRNA sequences were analyzed here with the quantitation for the subcellular distribution of YAP and pYAP shown underneath (n=6). Scale bar is 10 µm.

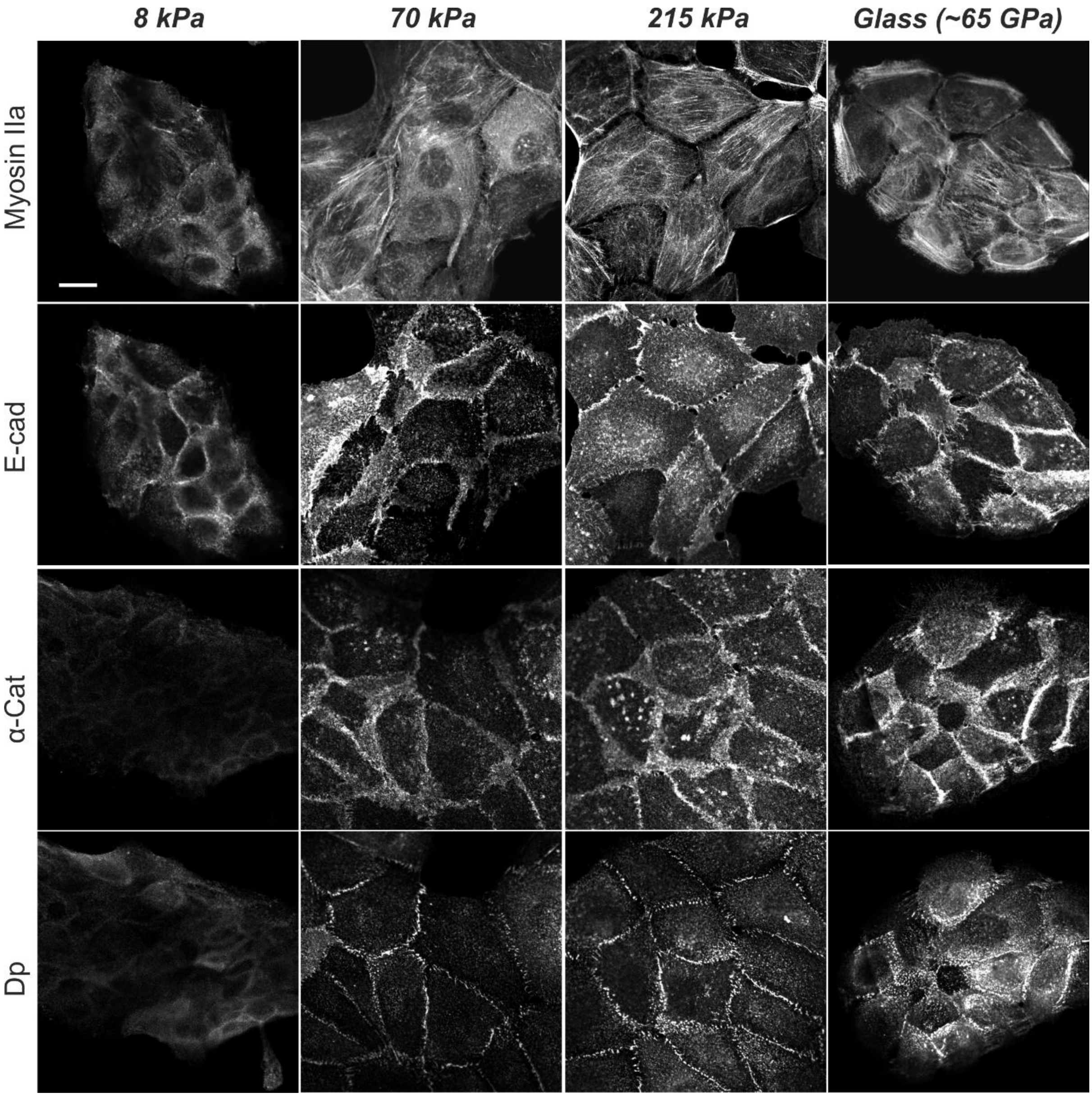

FIGURE S8. E-cadherin and Myosin IIa exhibit increased expression in response to substrate stiffness. Confocal images of HaCaT cells labeled for the indicated junctional proteins. Cells were seeded on collagen type I coated polyacrylamide hydrogels of varying stiffness. A general trend of correlation between protein expression and substrate stiffness was shown. In addition, there were more stress fibers in Myosin IIa staining in cells seeded on stiffer substrates, such as glass and hydrogels with 215 kPa. In contrast, diffuse cytoplasmic Myosin IIa staining was shown on softer hydrogels with 8 kPa and 70 kPa, respectively. Scale bar is 10 μm.

References

[1] P.P. Chen, W.J. Li, Y. Wang, S. Zhao, D.Y. Li, L.Y. Feng, X.L. Shi, H.P. Koeffler, X.J. Tong, D. Xie, Expression of Cyr61, CTGF, and WISP-1 correlates with clinical features of lung cancer, PLoS. One. 2 (2007) p.e534.
